# Evaluation of health status in broilers fed with a mixture of Dayak onion extract and *Lactobacillus acidophilus*

**DOI:** 10.5455/javar.2023.j678

**Published:** 2023-06-30

**Authors:** Iis Yuanita, Lisnawaty Silitonga, Nyoman Suthama

**Affiliations:** 1 Animal Science Study Program, Faculty of Agriculture, University of Palangka Raya, Palangka Raya, Indonesia; 2 Departement of Animal Science, Faculty of Animal and Agricultural Sciences, Diponegoro University, Semarang, Indonesia

**Keywords:** Feed additive, hematological, Lactobacillus acidophilus, lymphoid organs

## Abstract

**Objective:**

The feeding effects of DoLa (a combination of Dayak onion extract and probiotic *Lactobacillus acidophilus*) on hematological indices and lymphoid organs as indicators of broiler health status were evaluated in the present study.

**Materials and Methods:**

192 1-day-old unsexed broilers of the CP 707 strain with a body weight of 46.43 ± 1.65 gm were randomly divided into 4 dietary treatments with 6 replications. The dietary treatments applied were basal diet (BD) as a control with a code of DoLa0, BD + 0.1% DoLa (DoLa1), BD + 0.2% DoLa (DoLa2), and BD + 0.3% DoLa (DoLa3). The parameters monitored included hemoglobin (Hb), red blood cell (RBC), heterophile (H), lymphocyte (L), white blood cell (WBC), heterophile-lymphocyte (H/L) ratio, the lymphoid organs (bursa Fabricius, spleen, and thymus) relative weight, as well as carcass weight.

**Results:**

The results indicated a significant improvement in WBC, L, and carcass weight (*p* < 0.05) as the feeding level of DoLa increased while the H and H/L ratio decreased. However, the dietary inclusion of DoLa did not affect the lymphoid organs’ relative weight, RBC, and Hb concentrations.

**Conclusion:**

The mixture at 0.3% significantly improved health status through the indicators of hematological indices, lymphoid organs, and carcass weight of broilers.

## Introduction

Broilers are meat-producing animals with a faster growth rate than other poultry. The fast-growing chickens reared in the tropical region require feed additives to promote growth and immunity. Antibiotics have been utilized to boost the growth and productivity of flocks as well as combat the effects of disease and stressors on their health. Antibiotics used as growth promoters in poultry have been banned in numerous countries globally because of their detrimental effects on health and food safety, despite having been utilized previously. This is primarily due to the proliferation of pathogenic bacteria that have developed antibiotic resistance and the detection of antibiotic residues in animal products. Health preservation and disease prevention in animals are affected by various factors, with diet playing a crucial role. Antioxidants are one of many dietary factors that play a significant role in animal survival, health maintenance, productivity, and reproductive performance. The function of antioxidant substances is important to overcome the potential formation of free radicals during metabolic processes in chickens reared under hot climate exposure.

Various natural feed additives have been studied to enhance poultry health and improve feed efficiency. These additives offer diverse health benefits, each with its own biological function, such as serving as prebiotics, antioxidants, immunostimulants, or antimicrobial activity [1,2]. Antioxidants are essential for both nutrition and production performance in poultry. Similarly, Davani-Davari et al. [3] stated that using probiotics in poultry feed has also been shown to improve enzyme secretion and balance intestinal microbes. Cholis et al. [4] found that broilers fed with probiotic *Lactobacillus* sp. demonstrated a higher population of intestinal lactic acid bacteria and reduced total coliform, indicating improved bacterial balance. Similarly, Purbarani et al. [5] observed this improvement in native chickens administered a combination of *Lactobacillus* sp. and inulin.

Dayak onion (*Eleutherine palmifolia*) has many common names, such as berlian, sabrang, or tiwai onion. Wang et al. [6] reported that this plant belongs to the genus Iridaceae and produces bulbs with high levels of active compounds, namely phenolic and flavonoid contents. Phenolic compounds, as well as flavonoids, act as potent antioxidants and possess antibacterial properties [7,8], anti-inflammatory, immunomodulatory [9,10], antimicrobial, anthelmintic [11–13], and digestion-stimulating substances [14]. Puupponen-Pimia et al. [15] reported that some flavonoid derivatives, including phenolic acid, do not inhibit the growth of lactic acid bacteria. According to Puupponen-Pimia et al. [15], flavonoid derivatives such as phenolic acid can inhibit the growth of Gram-negative bacteria, such as *Salmonella *sp*.* and *Escherichia coli*. Mabrok et al. [16] reported that aside from being a source of antioxidant compounds, onion extract also contains prebiotics such as fructooligosaccharides and inulin at levels of 22.96 and 15.59 gm/100 gm dry matter, respectively.

According to Sapsuha et al. [17], Wulandari et al. [18], and Cholis et al. [4], *Lactobacillus *is a probiotic that is frequently utilized in broilers*. *Furthermore, Shoeib and Madian [19] and Sugiharto et al. [20] reported that an example of this probiotic is *Lactobacillus acidophilus*, which naturally resides in the animals’ digestive tract. Sugiharto [1] reported that it ferments simple carbohydrates to create lactic acid, which reduces the digestive tract’s pH and restrains pathogenic bacteria growth. Setyaningrum et al. [21], Cholis et al. [4], and Purbarani et al. [5] have shown that the short-chain fatty acid production from the fermentation of dietary low molecular-weight carbohydrates by *Lactobacillus *sp*.* can lead to a decrease in coliform growth by reducing intestinal pH. According to research, probiotic bacteria are thought to act as producers of antioxidants. Consequently, administering probiotic bacteria is anticipated to enhance the health and growth of broilers, as evidenced by hematological indicators.

Further investigation is required to determine the impact of a combination of Dayak onions with bacterial species as probiotics, in addition to their positive effect. In a previous study, a mixture of *L. acidophilus* and Dayak onion extract was evaluated. The optimal results were obtained with 75% Dayak onion extract and 10^8^ cfu/ml of *L.* *acidophilus*. This exhibited improved antioxidant activity, total phenolic content, and *Lactobacillus* liveability test results [22]. However, the dietary effect of supplementation on the health status of commercial broilers requires further examination. Hematological parameters serve as dependable indicators of the animal’s health status, and maximal growth can be achieved through their evaluation. Therefore, this study aims to assess the synergistic use of Dayak onion extract and *L. acidophilus* as antioxidant, prebiotic, and probiotic additives in the diet to enhance the boilers’ health performance. The results are expected to produce a more effective and valuable combination for the health status and carcass production of broilers.

## Materials and Methods

### Ethical approval

The research was conducted in accordance with the Guidelines for Ethical Conduct of Animal Care and Use in Research, followed by Directive Number 59-04/A-10/KEP-FPP of the Animal Ethics Committee, Faculty of Animal and Agriculture, University of Diponegoro.

### Preparation of the mixture 

To prepare the onion extract, the onion bulbs were divided into layers, thinly sliced, and air-dried under ambient conditions. They were smashed using a blender, then extracted with a methanol solution at a ratio of 1:4 (w/v) for 3 days. The extracted liquid was agitated at least once daily beginning on the fourth day and, at the end of the day, filtered using Whatman No. 1. Subsequently, with a rotational vacuum evaporator, the extracted liquid was evaporated to obtain a paste form, which was then stowed at refrigeration temperature for subsequent usage. The extraction method used was based on the procedure of Febrinda et al. [23].

The Biotechnology Laboratory at Gadjah Mada University provided pure, isolated *L. acidophilus *(FNCC 0051). In order to achieve *L. acidophilus*’ desired population (10^8^ cfu/ml), a culturing process was conducted at the Laboratory of Animal Nutrition Physiology and Biochemistry at Diponegoro University. The mixture levels were established using data from a prior *in vitro* study, which assessed three levels of Dayak onion extract (25%, 50%, and 75%) as well as three counts of *L.* *acidophilus* (10^6^, 10^7^, and 10^8^ cfu/ml). The most effective was discovered to be 75% Dayak onion extract and 10^8^ cfu/ml *L. acidophilus*, as determined by *Lactobacillus* liveability tests, antioxidant activity, total phenolic, and flavonoid content. Consequently, a unified formulation called DoLa was created with 75% Dayak onion extract in sterile water and 10^8^ cfu/ml* L. acidophilus* bacteria in a 1:1 (v/v) ratio. DoLa was used as the treatment material.

### Experimental animal and diet 

There are 192 unsexed 1-day-old broilers with an initial body weight of 46.43 ± 1.65 gm and CP 707, which were randomized into 4 dietary treatments with 6 replications each. Each replication of the experiment included eight broilers housed in litter-floored cages measuring 1 × 1 m. The broilers were nourished with an *ad libitum* diet and unrestricted access to drinking water until they reached 42 days of age. Samples were fed a commercial diet containing 3,070 kcal/kg ME, 20.5% CP, 5% fat, 0.9% Ca, and 0.6% P for the first seven days. Subsequently, a basal diet (BD) was prepared in compliance with the requirements specified in Table 1 of the Indonesian National Standards. From days 1 to 21, broilers were fed *ad libitum* with a starter diet, after which they were given a grower diet until day 42. The dietary treatments were created by adding DoLa at 0%, 0.1%, 0.2%, and 0.3% into the BD, with the codes DoLa0, DoLa1, DoLa2, and DoLa3, respectively.

### Sampling and parameters measurement 

The performance of broilers was measured by carcass weight. On day 42, three samples from each replication were selected at random. To obtain the relative weight of lymphoid organs, carcass weight, and whole meat samples, one broiler was slaughtered. Meanwhile, the remaining two broilers were used for collecting blood samples, which were extracted via the wing vein using a 3 ml syringe. It is transferred into properly labeled and sterilized tubes containing an anticoagulant (ethylenediamine tetra acetic acid). The samples were subjected to hematological analysis using a hematology analyzer according to the manufacturer’s instructions. The analysis consisted of lymphocytes (L), hemoglobin (Hb), heterophiles (H), white blood cells (WBCs), and red blood cells (RBCs).

**Table 1.** Composition of BD.

Ingredient	Composition (%)	
	Starter (<21 days)	Finisher (22–42 days)
Maize	56	55
Brand	10	16
Soybean meal	24	20
Meat bone meal	9.5	8
CaCO_3_	0.25	0.5
Mineral and vitamin premix^a^	0.25	0.5
Total	100	100
Nutrient content^b^		
Metabolizable energy, kcal/kg	2900	3042
Crude protein, %	20	18
Crude fiber, %	4.42	4.92
Ether extract, %	3.06	3.39
Ash, %	6.89	6.86
Calsium, %	1.01	1.08
Phosphor, %	0.64	0.63
Metionin, %	0.34	0.32
Lysine, %	1.09	1
Arginine, %	1.37	1.24

a Content per kg: calcium 32.5%, phosphor 1%, iron 6 gm, mangan 4 gm, iodine 0.075 gm, copper 0.3 gm, zinc 3.75 gm, vitamin B12 0.5 mg, vitamin D3 50,000 IU.

b Based on analyzed value at the Laboratory of Animal Nutrition and Feed Science, Faculty of Animal and Agricultural Sciences, Diponegoro University.

## Results 

There were no significant differences (*p* > 0.05) in the concentrations of RBC and Hb among all treatment groups at 42 days of age. However, the levels of WBC, H, L, heterophile-lymphocyte (H/L) ratio, and carcass weight were significantly affected by DoLa treatments (Table 2). The concentrations of WBC, L, and carcass weight significantly increased (*p* < 0.05), but H and H/L ratio decreased with the high level of feeding DoLa. The total WBC (×10^9^/l) of 77.25 in DoLa3 was significantly higher compared to other treatment groups. The results obtained varied from the highest value of 77.25 in DoLa3 to the lowest of 50.25 (10^9^/l) in DoLa0. Among all treatments, the carcass weight was found to be the highest in DoLa3, and there was a significant statistical difference (*p* < 0.05). On the other hand, DoLa0 had the lowest value.

As shown in Table 3, the relative weights of the thymus, spleen, and bursa Fabricius did not differ significantly (*p *< 0.05) between treatments. The bursa Fabricius, thymus, and spleen relative weights of DoLa treatments were 0.07%–0.15%, 0.19%–0.28%, and 0.11%–0.14%, respectively. The relative thymus weight was below the normal size, while that of the spleen and bursa Fabricius was within the normal range. The normal size of the bursa Fabricius and thymus of broilers aged 42 days was 0.09% and 0.48% of live weight, respectively [24], and the spleen ranged from 0.07% to 0.26% [17,25].

## Discussion

### Hematological indices and carcass weight 

According to the findings, no significant difference was observed (*p* > 0.05) between dietary treatments regarding RBC and Hb. While there was only a minor improvement in both RBC and Hb, the result of feeding DoLa was not statistically significant. The slight improvement in RBC due to the addition of DoLa may be attributed to its bioactive components, such as phenolics and flavonoids, that act as antibacterial agents, anti-inflammatory agents, immunomodulatory agents, and antimicrobial agents, as stated by Scalbert et al. [7] and Vauzour et al. [8]. The concentrations of RBC and Hb in all treatment groups were still within the acceptable ranges for normal chickens. The range of normal hematological values of RBC and Hb for broilers was 2.5–3.5 × 10^6^/μl and 10.20 gm/dl, respectively, as supported by El-Latif et al. [26] and Oloruntola et al. [27]. The slightly improved RBC count and Hb concentration of birds fed DoLa in this study suggest the supplements under study enhanced the adequate ingestion and absorption of essential nutrients that are needed in the processes involved in erythropoiesis.

**Table 2.** Hematological indices and carcass weight.

Parameters	Dietary treatments				
	DoLa0	DoLa1	DoLa2	DoLa3	*p* value
RBC (10^12^/l)	2.23 ± 0.43	2.53 ± 0.18	2.26 ± 0.34	2.55 ± 0.15	0.15
WBC (10^9^/l)	50.25 ± 17.65^b^	59.00 ± 4.94^b^	65.00 ± 9.22^ab^	77.25 ± 15.18^a^	0.01
Hb (gm/dl)	9.67 ± 1.21	10.83 ± 0.68	9.92 ± 1.71	10.92 ± 0.66	0.18
Heterophile (10^9^/l)	11.42 ± 4.59^a^	7.25 ± 2.64^b^	7.50 ± 2.98^b^	5.75 ± 1.81^b^	0.03
Lymphocyte (10^9^/l)	38.75 ± 14.82^b^	49.75 ± 4.95^b^	48.42 ± 4.39^b ^	65.08 ± 14.79^a^	0.005
H/L	0.28 ± 0.08^a^	0.12 ± 0.04^b^	0.16 ± 0.08^a^	0.11 ± 0.05^b^	0.0098
Carcass weight (gm)	747.33 ± 53.04^b^	752.83 ± 23.10^b^	760.5 ± 29.26^b^	861.5 ± 56.21^a^	0.005

^a,b^Means within row followed by different superscript differ significantly (*p* < 0.05), DoLa0 (BD), DoLa1 (BD added with 0.1% DoLa), DoLa2 (BD added with 0.2% DoLa), DoLa3 (BD added with 0.3% DoLa).

**Table 3.** Relative weight of lymphoid organs.

Lymphoid	Dietary treatments				
organ	DoLa0	DoLa1	DoLa2	DoLa3	*p* value
Bursa Fabricius (%)	0.11 ± 0.04	0.15 ± 0.13	0.09 ± 0.03	0.07 ± 0.03	0.31
Thymus (%)	0.20 ± 0.08	0.19 ± 0.09	0.28 ± 0.19	0.25 ± 0.12	0.64
Spleen (%)	0.14 ± 0.02	0.14 ± 0.04	0.12 ± 0.01	0.11 ± 0.01	0.41

DoLa0 (BD), DoLa1 (BD added with 0.1% DoLa), DoLa2 (BD added with 0.2% DoLa), DoLa3 (BD added with 0.3% DoLa).

At the end of the 6-week experiment, the effects of DoLa on the WBC count, heterophile (H), lymphocyte count (L), and heterophile to lymphocyte ratio (H/L) of broilers were measured (Table 2). The results indicated that total WBC and L counts were higher and H concentrations were lower in broilers fed with DoLa3. L plays a critical role in regulating the immune system, while H serves as the primary defense against infections [28]. The higher H count in the DoLa0 group can be due to the function of maintaining healthy conditions, specifically attacking endogenous pathogenic bacteria, which is also associated with the lowest WBC count. A previous investigation indicated that the highest amount of coliform, an endogenous pathogen, was found in birds given a diet without additive probiotics of *L. acidophilus* [22,29]. In this study, it can be stated that the profile of H may signify the presence of numerous pathogens due to the administration of DoLa0. According to Yuanita et al. [22], the coliform total in the ileal digesta of broilers was significantly lower in DoLa1, DoLa2, and DoLa3, with values of 4.17 ± 0.47, 4.74 ± 0.38, and 3.65 ± 0.17 log CFU/gm, respectively, than the control group, which had 6.96 ± 0.07 log CFU/gm [22]. Since DoLa has an antioxidant property responsible for maintaining WBC counts, its additive treatment will positively improve hematological conditions.

The leukocytes circulating in the blood significantly function in the immune system and are responsible for the phagocytosis of infectious antigens. In this study, supplementation of DoLa into broilers’ diets increased leukocyte (WBC) concentration. The dietary phytochemicals used were well-known antioxidants, and their activity had been demonstrated *in vivo* and *in vitro* studies [22,30]. However, El-Latif et al. [26] and El-Katcha et al. [31] stated that the onion or garlic supplementation in broilers’ diet did not affect WBC integrity. Leukocytes are an active unit in the body’s defense system. The provision of antioxidant substances increased lymphocyte counts, thereby enhancing the immune status of chickens. The synergistic work of DoLa (antioxidant and probiotic action) can maintain microbial balance and improve the immune system of broiler chickens.

The H/L ratio provides a stress indication in livestock, including broilers. As presented in Table 2, chickens with no DoLa supplementation have the highest H/L ratio. This stress can be caused by exposure to hot environmental conditions in the tropical country since chickens were maintained in an open house with temperatures ranging from 27°C to 32°C. However, the improved health status with higher WBC levels and a lower H/L ratio was very helpful for the effectiveness of nutrition metabolism. This was beneficial for boosting broilers’ growth and producing better performance, as indicated by higher muscle production. The improved production performance of broilers fed diets supplemented with DoLa can be attributed to enhanced gut health, which is further evidenced by greater physical body resistance and a reduced H/L ratio. The healthier the broilers, the more efficiently nutrients are utilized, resulting in improved carcass weight (*p* < 0.05).

Studies indicate that flavonoids have the ability to handle mucosal as well as cellular immunity while also modulating health’s endocrine and circulatory markers. Therefore, dietary supplementation with flavonoids can improve broilers’ immunity and health, as supported by the results of Yuanita et al. [22,30]. The plant-based flavonoids have been found to exhibit antioxidant, anti-inflammatory, antibacterial, and immunostimulating properties [32], which can be explained by the health effects of broilers observed.

### Lymphoid organ relative weight

The lymphoid organs’ size or relative weight indicates antibody formation. The thymus and bursa Fabricius can shrink when overworked to produce antibodies to support the immune system. The bursa Fabricius undergoes depletion and decreases relative weight as it works harder to form antibodies. Additionally, the thymus experiences rapid atrophy, which leads to a decrease in size. Meanwhile, when broilers are affected by the disease, the spleen enlarges as it works excessively hard to form antibodies, as reported by Landung et al. [33]. Based on the lymphoid organs relative weight in Table 3, it can be stated that the administration of DoLa treatment tended to promote good health and prevent infection in broilers. This was indicated by the spleen size remaining stable while the bursa Fabricius and thymus did not significantly decrease. The effects of Dayak onion extract and the probiotic *L. acidophilus*, which contain flavonoid and phenolic antioxidants, may cause this condition. In this study, the extracted total phenolic content of the Dayak onion was 16,284 ppm (16.28 gm/kg). The obtained results were higher compared to the findings of Aditya et al. [34], Kavalcova et al. [35], and Bouba et al. [36], which were 0.39, 0.397, and 0.62 gm/kg. The stimulatory effects of some herbs on *L. acidophilus* [37] have been reported, suggesting a positive relationship between the antioxidant activity of the mixture and the health condition of broilers. Therefore, the good health status and good antibody production in broilers fed with DoLa can be inferred from the lymphoid organs’ relative weight.

## Conclusion

The broilers’ health status was improved by the mixture of Dayak onion extract and probiotic *L. acidophilus*, specifically at a concentration of 0.3% (DoLa3), as demonstrated by the hematological indices and lymphoid organs’ relative weight.

## List of abbreviations

H, heterophile; Hb, hemoglobin; H/L, heterophile-lymphocyte ratio; L, lymphocyte; RBC, red blood cell; WBC, white blood cell.

## References

[1] Sugiharto (2016). Role of nutraceuticals in gut health and growth performance of poultry. J Saudi Soc Agric Sci.

[2] El-Sabrout K, Khalifah A, Mishra B (2023). Application of botanical products as nutraceutical feed additives for improving poultry health and production. Vet World.

[3] Davani-Davari D, Negahdaripour M, Karimzadeh I, Seifan M, Mohkam M, Masoumi SJ (2019). Prebiotic: definition, types, sources, mechanisms, and clinical applications. Foods.

[4] Cholis MA, Suthama N, Sukamto B (2018). Feeding microparticle protein diet combined with *Lactobacillus* sp. on existence of intestinal bacteria and growth of broiler chickens. J Indonesian Trop Anim Agric.

[5] Purbarani SA, Wahyuni HI, Suthama N (2019). Dahlia inulin and *Lactobacillus* sp. in step down protein diet on villi development and growth of KUB chickens. Trop Anim Sci J.

[6] Wang H, Cui Y, Zhao C (2010). Flavonoids of the genus *Iris* (Iridaceae). Mini-Rev Med Chem.

[7] Scalbert A, Manach C, Morand C, Rémésy C, Jiménez L (2005). Dietary polyphenols and the prevention of diseases. Crit Rev Food Sci Nutr.

[8] Vauzour D, Rodriguez-Mateos A, Corona G, Oruna-Concha MJ, Spencer JPE (2010). Polyphenols and human health: prevention of disease and mechanisms of action. Nutr.

[9] Grashorn MA (2010). Use of phytobiotics in broiler nutrition—an alternative to infeed antibiotics. J Anim Feed Sci.

[10] Muthusamy N, Sankar V (2015). Phytogenic compounds used as a feed additive in poultry production. Int J Environ Sci Tech.

[11] Abbas M, Saeed F, Anjum FM, Afzaal M, Tufail T, Bashir MS (2017). Natural polyphenols: an overview. Int J Food Prop.

[12] Pietrzyk L (2016). Food properties and dietary habits in colorectal cancer prevention and development. Int J Food Prop.

[13] Manafi M (2015). Comparison study of a natural non-antibiotic growth promoter and a commercial probiotic on growth performance, immune response, and biochemical parameters of broiler chicks. J Poult Sci.

[14] Ganguly S (2017). Herbal antioxidant agents, and its pharmacological and medicinal properties.

[15] Puupponen-Pimia R, Nohynek L, Meier C, Heinonen M, Hopia A, Caldentey KM (2001). Antimicrobial properties of phenolic compounds from berries. J Appli Microb.

[16] Mabrok H, Soliman M, Mohammad M, Hussein L (2018). HPLC profiles of onion fructooligosaccharides and inulin and their prebiotic effects on modulating key markers of colon function, calcium metabolism and bone mass in rat model. J Biochem Phys.

[17] Sapsuha Y, Hasan S, Nur A (2023). Survivability of *Lactobacillus plantarum *in nutmeg (*Myristica fragrans *Houtt) flesh extract and its effect on the performance of broiler chicken. J Adv Vet Anim Res.

[18] Wulandari LT, Suthama N, Sukamto B (2018). Blood parameters and productivity of broilers fed ration composed of microparticle protein with the addition of *Lactobacillus* sp. J Indonesian Trop Anim Agric.

[19] Shoeib HK, Madian AH (2002). A study of the effect of feeding diets containing probiotics (Pronifer and Biogen) on growth performance, intestinal flora and haematological picture of broiler chicks. Assiut Vet Med J.

[20] Sugiharto Yudiarti T, Isroli Widiastuti E, Wahyuni HI, Suprijatna E (2018). The potential of *Bacillus* strains isolated from the rumen content of dairy cows as natural antibacterial and antioxidant agents for broilers. J Indonesian Trop Anim Agric.

[21] Setyaningrum S, Yunianto VD, Sunarti D, Mahfudz LD (2019). The effect of synbiotic (inulin extracted from gembili tuber and *Lactobacillus plantarum*) on growth performance, intestinal ecology and haematological indices of broiler chicken. Livest Res Rural Dev.

[22] Yuanita I, Sunarti D, Wahyuni HI, Suthama N (2020). A combination of Dayak onion (*Eleutherine palmifolia*) extract and *Lactobacillus acidophilus* on antioxidant capacity and intestinal bacteria in broiler chickens. IOP Conf Ser Earth Environ Sci.

[23] Febrinda AE, Astawan M, Wresdiyati T, Yuliana ND (2013). Antioxidant and alpha-glucosidase inhibitor properties of Bawang Dayak bulb extracts. J Teknol Ind Pang.

[24] Toghyani M, Tohidi M, Gheisari AA, Tabeidian SA (2010). Performance, immunity, serum biochemical and hematological parameters in broiler chicks fed dietary thyme as alternative for an antibiotic growth promoter. Afr J Biotechnol.

[25] Sugiharto S, Yudiarti T, Isroli I, Widiastuti E, Wahyuni HI (2018). Hematological parameters and selected intestinal microbiota populations in the Indonesian indigenous crossbred chickens fed basal diet supplemented with multi-strain probiotic preparation in combination with vitamins and minerals. Vet World.

[26] El-Latif SAA, Toson MA, Mehany HA (2019). Effect of dietary onion, garlic, red pepper and anise as natural feed additives on some hematological studies of Japanese quail chicks. Acta Sci Nutr Health.

[27] Oloruntola OD, Ayodele SO, Adeyeye SA, Oloruntola DA, Osowe CO, Fasuhami OS (2022). Broiler chickens’ growth, haematological indices, guts microbiota, carcass and meat analysis in response to dietary supplementation with *Anacardium occidentale *leaf powder and a mix of prebiotic, probiotic and acidifier. Poult Stud.

[28] Latimer KS, Bienzle D, Weiss DJ, dan Wardrop KJ (2010). Determination and interpretation of the avian leuokogram. Schalm’s veterinary hematology.

[29] Saputra YA, Suthama N, Sukamto B (2020). Feeding diets composed of low level microparticle protein derived from fish and soybean meals and using organic calcium added with *Lactobacillus acidophilus* or citric acid on intestinal condition and performance of broilers. Livest Rural Res Develop.

[30] Yuanita I, Sunarti D, Wahyuni HI, Suthama N (2019). Feeding Dayak onion (*Eleutherine palmifolia*) extract and *Lactobacillus acidophilus* mixture on blood biochemicals, meat quality characteristics and growth performance in broiler chickens. Livest Rural Res Develop.

[31] El-Katcha MI, Soltan MA, Sharaf MM, Hasen A (2016). Growth performance, immune response, blood serum parameters, nutrient digestibility and carcass traits of broiler chicken as affected by dietary supplementation of garlic extract (*Allicin*). Alex J Vet Sci.

[32] Kamboh AA, Arain MA, Mughal MJ, Zaman A, Arain ZM, Soomro AH (2015). Flavonoids: health promoting phytochemicals for animal production: a review. J Anim Health Prod.

[33] Landung DC, Mahfudz LD, Suthama N (2013). Effect of flour red guava fruit (*Psidium guajava* L.) in the ration to the development of the small intestine and the growth of broiler chickens. Anim Agric J.

[34] Aditya S, Ahammed M, Jang SH, Ohh SJ (2017). Effects of dietary onion (*Allium cepa*) extract supplementation on performance, apparent total tract retention of nutrients, blood profile and meat quality of broiler chicks. Asian Australas J Anim Sci.

[35] Kavalcova P, Bystricka J, Tomas J, Karovicova J, Kuchtova V (2014). Evaluation and comparison of the content of total polyphenols and antioxidant activity in onion, garlic and leek. Potravinarstvo.

[36] Bouba A, Toua V, Nkouam GB (2012). Effect of solar and electric drying on the content of the phenolic compounds and antioxidant activity of three varieties of onion (*Allium cepa* L.). Inter J Biol Pharm Allied Sci.

[37] Zhou Q, Wang S, Yang G, Zhao W, Li H (2016). Development and evaluation of a herbal formulation with anti-pathogenic activities and probiotics stimulatory effects. J Integr Agric.

